# Influence of Herbal Fillers Addition on Selected Properties of Silicone Subjected to Accelerated Aging

**DOI:** 10.3390/polym15010042

**Published:** 2022-12-22

**Authors:** Sara Sarraj, Małgorzata Szymiczek, Sebastian Jurczyk

**Affiliations:** 1 Department of Theoretical and Applied Mechanics, Silesian University of Technology, Konarskiego 18A, 44-100 Gliwice, Poland; 2 Łukasieiwcz Research Network—Institute for Engineering of Polymer Materials and Dyes, M. Sklodowska-Curie 55, 87-100 Toruń, Poland

**Keywords:** biocomposites, herbs, polyphenols, organosilicon polymer

## Abstract

This work aims to assess the impact of the type and percentage of powdered herbs on selected properties of silicone-based composites. The matrix was an addition cross-linked platinum-cured polydimethylsiloxane. The fillers were powdered thyme and sage, which were introduced at 5, 10, and 15 wt.%. The introduced fillers differed in composition, morphology, and grain size. The grain morphology showed differences in the size and shape of the introduced fillers. The qualitative and quantitative assessment resulting from the incorporation was conducted based on tests of selected properties: density, wettability, rebound resilience, hardness, and tensile strength. The incorporation slightly affected the density and wettability of the silicone. Rebound resilience and hardness results differed depending on the filler type and fraction. However, tensile strength decreased, which may be due to the matrix’s distribution of fillers and their chemical composition. Antibacterial activity evaluation against *S. aureus* proved the bacteriostatic properties of the composites. Accelerated aging in PBS solution further deteriorated the mechanical properties. FTIR and DSC have demonstrated the progressive aging of the materials. In addition, the results showed an overall minimal effect of fillers on the silicone chemical backbone and melting temperature. The developed materials can be used in applications that do not require high mechanical properties.

## 1. Introduction

Unlike other polymeric materials, silicones have unique properties, such as flexibility at a wide range of temperatures, chemical stability, gas and drug permeability, and low surface tension [1]. These properties manifest in high biodurability and biocompatibility. In the medical field, these materials have found application in short and long-term implants (e.g., urinary catheters [2], voice prostheses [3], maxillofacial prostheses [4], airway stents [5], and wound dressings [6]), as well as medical devices and instruments. However, these materials are characterized by low antimicrobial properties, making them prone to colonization by bacteria or fungi and forming resistant biofilms. This leads to progressive infection of the surrounding tissue, causing severe health problems.

Recent developments in medicine have heightened the need for biomaterials fulfilling not only the basic functional requirements, but also limiting the expansion of microbial colonies on their surface. Scientists are constantly seeking new means to improve these materials. Literature reports different ways of achieving better mechanical properties, improved antimicrobial properties, or both. The modifications can be either made to the structure of the silicone matrix, i.e., by incorporating fillers or by coating the material. Many works reported the favorable impact of coating the silicone surface with sophorolipids [7], R89 biosurfactant [8], nanogold [3], and silver nanoparticles [9] on the antimicrobial properties. However, this kind of modification mainly limits bacterial or fungal colonization and has little impact on the mechanical properties. On the other hand, mechanical properties can be influenced by incorporating graphene and carbon nanotubes [10,11], silver-zirconium phosphate and titanium oxide [12,13], titanium dioxide [14], zinc oxide [15], PMMA [16], or bioglasses [17,18], some of which improve the antimicrobial properties and biointegration of implants as well. Another way to reduce the adhesion of bacteria to the material is to modify the surface by interacting with chemical compounds, plasma or nanopattering, which allows for obtaining a specific surface topography [19,20,21].

Nevertheless, the modification methods mentioned above need chemical preparation before incorporation. This generates higher coats and often involves using harmful chemicals. With that in mind, researchers are turning towards acquiring composites incorporated with organic fillers owing to their adequate properties, such as availability, renewability, and simplicity of preparation, compared to inorganic fillers. Multiple works reported the changes in the silicone matrix after incorporating it with different organic materials. Some studies proved their favorable impact on mechanical properties [22,23], and others, the opposite [24]. The results varied depending on the filler type, fraction, and structure.

Another approach is to incorporate plant extracts highly rich in phytochemicals called polyphenols. Polyphenols are secondary metabolites chemical compounds naturally occurring in plants and herbs. They are responsible for defense against ultraviolet radiation and pathogens’ aggression and contribute to the plant’s color, flavor, and odor [25]. Maroufi et al. [26] successfully incorporated thyme extract into an electrospun polylactic acid and proved their favorable impact on the mechanical and biological properties. Multiple researchers achieved similar results using different herbal fillers [27,28,29,30]. In a different approach, researchers incorporated thyme in a powdered form. In one work [31], incorporating thyme had a beneficial influence on mechanical properties; other works stated the opposite [32]. The difference in the results is related to the varying filler size and matrix material.

In the case of materials modification, an essential factor is the analysis of their behavior and changes in characteristics during use. For this purpose, accelerated aging tests are carried out in simulated conditions similar to the working environment. The majority of these procedures are performed under elevated temperatures. This causes the formation of cross-linked structures. Water vapor reduces the molecular weight, and gamma radiation affects the oxidation of siloxane [33,34]. The degradation process depends on the chemical structures, the modification type, and the product manufacturing technology [35].

However, to the best of our knowledge, limited works study the impact of the direct incorporation of polyphenols-rich herbal fillers into an organosilicon polymer matrix, which the authors took upon themselves to investigate.

This paper will focus on the possibility of developing new silicone biocomposites incorporated with powdered herbal fillers and evaluating the changes occurring in the polymeric matrix. The assessment will be based on several physical, chemical, biological, and mechanical tests. Moreover, the materials will be subjected to accelerated aging in a solution simulating the human extracellular fluid.

## 2. Materials and Methods

### 2.1. Materials Preparation

For the purpose of this paper, an additional-crosslinking platinum-cured medical grade silicone Dragon Skin^TM^ 30 (based on the IR spectrum in Section 3.8 it was determined that the studied material is polydimethylsiloxane (PDMS)) was chosen as the composites’ matrix (Table 1). The chosen matrix is commonly used in medical applications, and this particular silicone has a skin safety certification. In order to improve the antibacterial properties, the material was modified with herbs.

**Figure 1:**
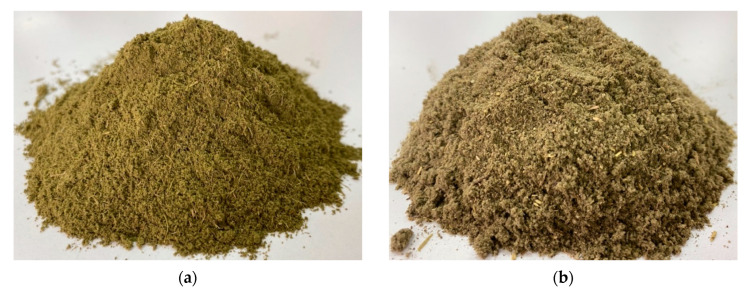
Fillers after drying and milling: thyme (**a**) and sage (**b**).

**Figure 2:**
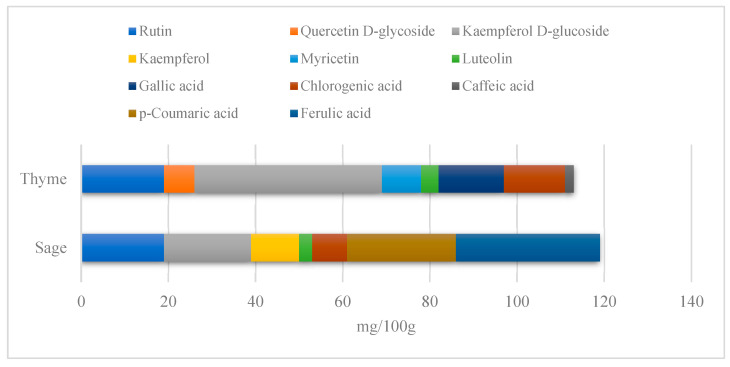
Polyphenolic compounds of thyme and sage. Adapted from [38].

We chose two commonly available herbs as modifiers: thyme (*Thymus vulgaris*) and sage (*Salvia officinalis*)—Figure 1—the composition of which is presented in Table 2 and Figure 2. The following criteria were the basis of herbs selection process:

- Similar polyphenolic compounds composition;
- Similar antimicrobial properties;
- Wide availability.

Although the herbs were purchased dried, they underwent additional drying for 1 h at 20 °C to eliminate any water residue without affecting the temperature-sensitive polyphenolic compounds. Next, the fillers were soaked in ethanol (90%) for 4 h and dried at 30 °C for 24 h. This procedure was carried out to dissolve oil compounds from the herbs, which disturb the curing of the silicone and affect the material’s mechanical properties. Later, the dried herbs were added to the premixed silicone base and catalyst (mass ratio 1:1) in different mass weight content (5, 10, and 15%) and mixed for 3 min at the speed of 150 rpm. After mixing, the compositions were vacuum deaerated for 4 min to eliminate any trapped air bubbles and achieve a homogenous non-porous structure. The materials were prepared using gravity casting. The curing time was 24 h at room temperature and an additional hour at 60 °C. After curing, thermal conditioning was conducted for 2 h at 80 °C. The materials codes and concentrations are presented in Table 3. Materials were prepared and tested at 20 ± 2 °C and 50% humidity.

### 2.2. Research of the Composites’ Properties

The research aim in the first stage was to determine the effect of fillers in the form of powdered herbs of thyme and sage on the basic properties of the tested materials. The studies of operating characteristics included the tests of:

- Grains’ size and morphology;
- Microscopic images of composites cross-section;
- Topography microscopy;
- Physicochemical properties (density, wettability, rebound resilience, Fourier transform infrared (FTIR) spectroscopy, and differential scanning calorimetry (DSC) curve analysis);
- Mechanical properties (rebound resilience, stress at break, and elongation at break);
- Biological tests (antibacterial activity).

All tests were carried out on samples prepared according to the procedure described in Section 2.1. Constant test conditions were also ensured—temperature 20 ± 2 °C, humidity 50 ± 2%, air-conditioned room.

#### 2.2.1. Grain Morphology

Grain size and distribution were determined by Laser Diffraction (LD) conducted on a Fritsch Analysette 22 Micro Tec Plus (FRITSCH GmbH, Idar-Oberstein, Germany). Grain morphology was visualized using scanning electron microscopy (SEM) conducted on Zeiss Supra 35 Microscope (Carl Zeiss AG, Oberkochen, Germany). About 5 g of prepared ground herbs was tested. The samples were sputtered with gold powder for 90 s, and the operating voltage was 10 kV. The morphology was observed under a magnification of 250× and 5000×.

#### 2.2.2. Samples Topography

The topography of the samples was visualized using a stereoscopic microscope Zeiss stereo discovery v12. A magnification of 100× was applied.

#### 2.2.3. Density

The density of the reference material and herbs–silicone composites were determined using an analytical balance equipped with a hydrostatic density measurement kit (Ohaus Adventurer Pro, OHAUS Europe GmbH, Greifensee, Switzerland) following the standard ISO 1183-1 [39]. The test was carried out on five samples of each material using the immersion method.

#### 2.2.4. Wettability

The samples’ surface wettability was determined by measuring the contact angle on SURFTENS UNIVERSAL goniometer (OEG Gesellschaft, Frankfurt, Germany). The measurements were conducted using the sessile drop method following the standard EN 828:2013 [40] using distilled water. Five drops, with a volume of 1 µL, were dropped on the surface of each test sample. The duration of one measurement was 60 s with a sampling frequency of 1 Hz.

#### 2.2.5. Fourier Transform Infrared Spectroscopy

FTIR spectra were obtained in attenuated total reflection (ATR) mode using IRSpirit FTIR spectrophotometer (Shimadzu Corporation, Kyoto, Japan). Spectra were collected over the mid-infrared range (4000–400 cm^−1^) with a resolution of 4 cm^−1^ and 20 scans. A background spectrum against air was collected prior to testing. The ATR diamond crystal was cleaned with ethanol after each test.

#### 2.2.6. Differential Scanning Calorimetry

DSC analysis of the reference samples and after accelerated aging was performed at temperature scan mode using a DSC 1 model differential scanning calorimeter (Mettler Toledo, Greifensee, Switzerland). Specimens of 20–21 mg were weighted in an XS105DU analytical balance (Mettler Toledo, Greifensee, Switzerland). Samples were hermetically closed in aluminum pans with lids (40 µL) (a new pan for each test was used). Samples were heated from −90 °C to 220 °C at a rate of 20 °C per minute and cooled at the same rate in a nitrogen atmosphere (60 mL min^−1^). DSC results were analyzed using the supplied software STARe Mettler Toledo. The test was carried out following the standard ISO 11357-1 [41].

#### 2.2.7. Rebound Resilience

To evaluate the prepared composites’ elasticity, a rebound resilience test was carried out using Schob machine (Heckert, Chemnitz, Germany). Test samples were mechanically conditioned with two impacts before testing. Each sample was tested three times in accordance with the standard ISO 4662 [42].

#### 2.2.8. Hardness

The samples’ hardness was measured using a Shore type A durometer (Zorn Stendal, Stendal, Germany). The test was carried out following the standard ISO 7619-1 [43] and consisted of five measurements of each material.

#### 2.2.9. Static Tensile Testing

**Figure 3:**
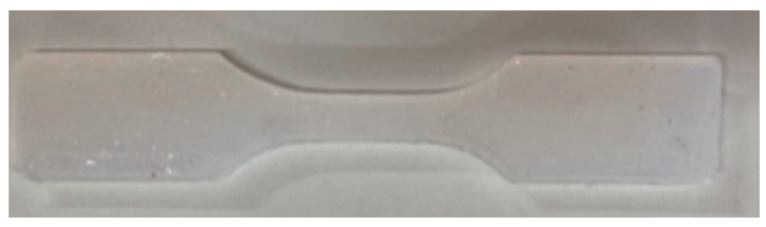
Tensile test specimens.

**Figure 4:**
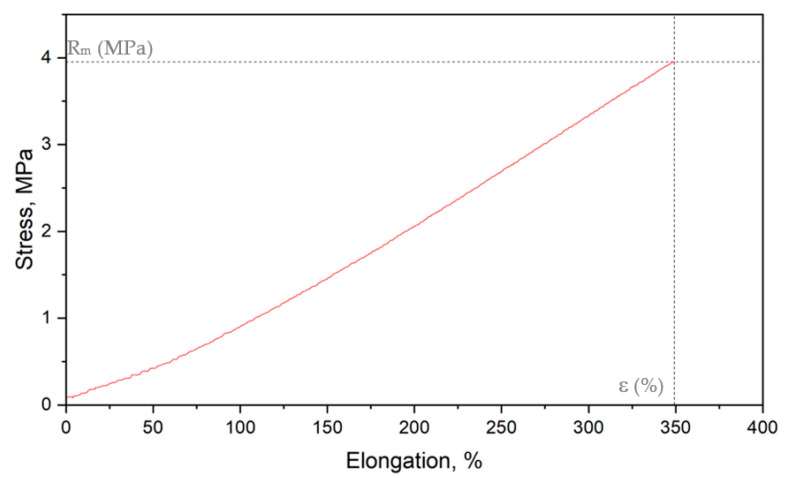
Stress–strain chart of the reference sample (Rm—stress at break, ε—elongation at break).

The static tensile test was performed using a Shimadzu kN10D testing machine (Shimadzu Corporation, Kyoto, Japan) following the standard ISO 527 [44]. The crosshead speed was 50 mm/min. The samples were 60 mm long and 4 mm wide along the measuring length (ISO 527-1–type 5-B (Figure 3)). Stress at break and elongation at break were determined based on the example diagram presented in Figure 4.

The tested properties of the unaged samples (the so-called reference samples) were used to determine the influence of aging on the qualitative and quantitative changes occurring in the material.

#### 2.2.10. Antibacterial Activity Assessment

The biological studies were performed for the highest filling as the matrix (silicone) was assumed to be approved for medical use. The highest degree of filling will have the greatest impact on changes in properties; therefore, only composites with 15% of powdered herbs were subjected to biological tests.

The samples were prepared, and the test was carried out following the standard ISO 22196 [45]. Three samples of each material (40 mm × 40 mm square dimensions) were tested against *Staphylococcus aureus* ATCC 6538 strain. The first step was to inoculate the strain onto Mueller Hinton agar and incubate it at 35 ± 1 °C for 24 h. Next, the test specimens were disinfected using ethanol and placed in Petri dishes. A cell suspension of *S. aureus*—1.1 × 10^6^ CFU/mL (colony forming unit/mL)—was prepared, and a 0.4 mL drop was placed onto the surface of the tested specimens. The samples were covered with 70 μm thick ethylene vinyl alcohol (EVOH) films. The Petri dishes were placed in a container with sterile water and incubated at 35 ± 1 °C for 24 h. In the next step, the samples were washed with 10 mL of D/E neutralizing broth, and decimal dilution was carried out. From each dilution and the control sample, 1 mL was transferred to a Petri dish filled with agar and filled with nutrient broth. Next, the plates were incubated at 35 °C for 24 h, after which bacterial colonies were counted. The reduction log was calculated based on the initial inoculum and subtracting the log_10_ count of the control group.

### 2.3. Accelerated Aging

In order to determine the changes in the material during use, aging was carried out under conditions simulating a biologically active environment. The composites were subjected to an accelerated degradation process by immersion in phosphate-buffered saline (PBS) solution simulating the human extracellular fluid, corresponding to approximate operating conditions. The samples were kept for 2 and 60 days at 70 °C in accordance with the standard ISO 10993-13 [46] in such a way as to ensure free fluid flow between the samples.

The degradation degree was assessed by microscopic observation and comparing the mechanical properties as well as the chemical and physical properties of the aged samples in reference to the native ones. The changes observed in the characteristic peaks of the FTIR spectra and the inflection of the DSC curves, as well as the values of stress at break, elongation at break, and hardness, allowed for the qualitative and quantitative assessment of the aging influence under the assumed conditions on the properties of the tested composites.

## 3. Results and Discussions

### 3.1. SEM Observations

**Figure 5:**
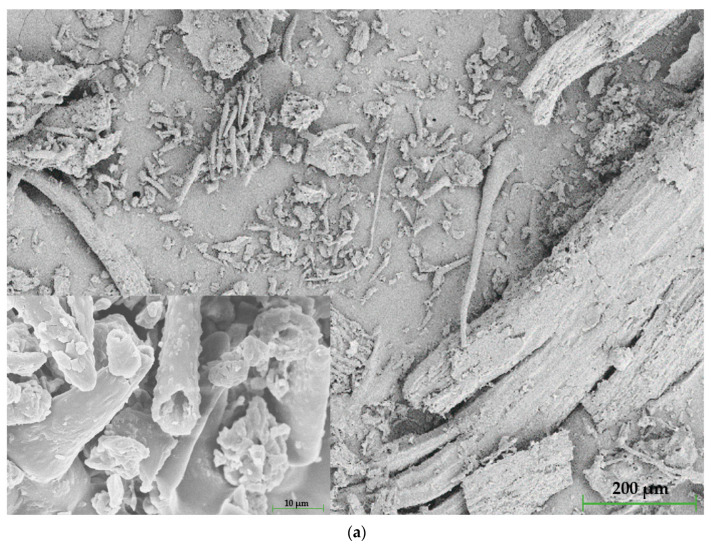

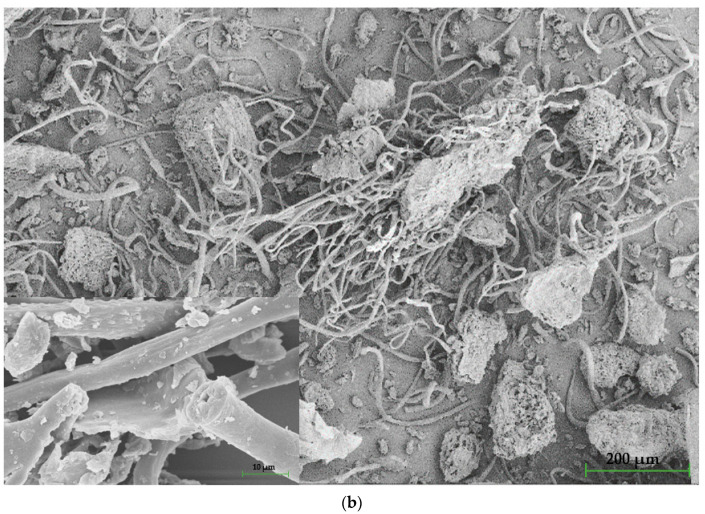
SEM micrographs of thyme—(**a**) and sage—(**b**).

The fillers’ grains shape is illustrated in Figure 5.

Both fillers have developed surfaces with structures of different sizes. In thyme micrographs (Figure 5a), the grains are characterized by bent, unbranched, needle-like trichomes (structures responsible for protection and odor) with a rough surface and visible stomas. At higher magnification, it can be observed that the trichomes are opened. This is related to the grinding process, due to which the trichomes release an odor characteristic of thyme. In addition, grains’ sizes vary from smaller, shorter structures to bigger, longer ones, which is proved in particle size analysis in Section 3.2. In addition, the grains exhibit extensive internal structures showing high porosity, affecting the matrix’s adhesion. Like thyme, sage’s grains are characterized by visible stomas and trichomes; however, they are longer and form a far-reaching net rather than multiple assemblages (Figure 5b). The differences between the fillers are mainly related to the nature of the plants and their individual behavior during the grinding process. It should be noted that despite the purchase of dried fillers and then subjecting to additional drying, the method used for drying fresh herbs, which may additionally affect the grinding behavior of the herbs, is unknown to the authors. The well-developed surface of the fillers is one of the main factors influencing the properties of the obtained composites. Moreover, thyme stalks are highly branched (after years, they become woody), and the leaves reach a size of up to 4 cm. Woody stems affect the properties due to increased hardness. In the case of sage, the leaves are mainly used and do not show any woody parts, which does not significantly affect the test results. Woody parts can act like a notch, affecting the results’ scatter [47].

### 3.2. Grain Size

**Figure 6:**
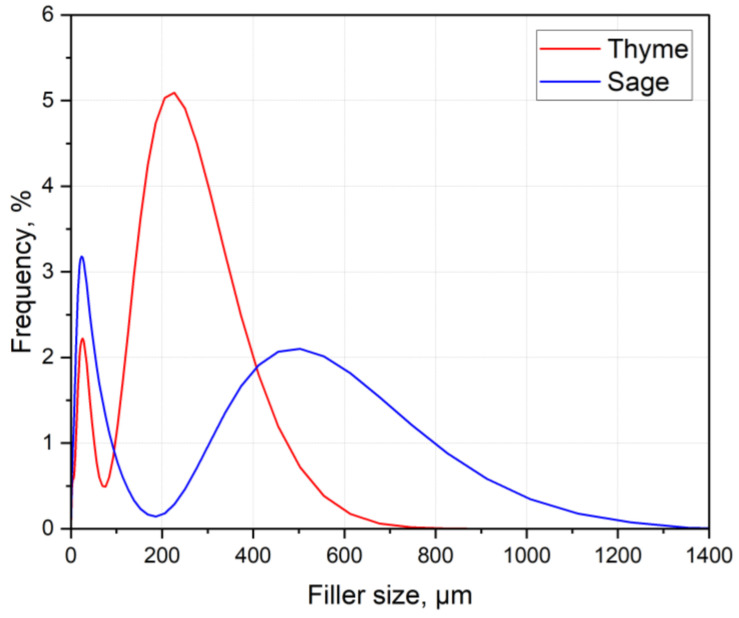
Particle size distribution of the fillers’ grains.

Particle size analysis results illustrate the fillers’ grain size and distribution based on five measurements of each material (Figure 6).

Both fillers show bimodal grain size distribution consisting of fine and coarse grains. Sage grains are characterized by a more uniform distribution between the largest and the smallest particles. In thyme’s case, the most common size is 227 µm (6.62%), while for sage, it is 23 µm (3.4%). However, the 90th percentile of thyme grains equals 281.73 µm, whereas, for sage, it equals 471.12 µm, making sage grains bigger than thyme by approx. 67%. The differences in the herbs’ grain distribution are attributed to their individual behavior during the grinding process. The grain distribution and morphology play an essential part in structure forming and, hence, properties’ shaping.

### 3.3. Density

**Figure 7:**
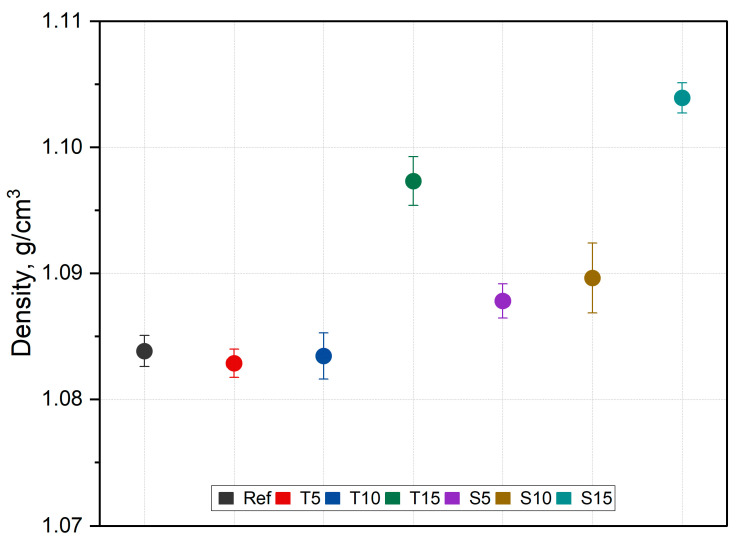
Density results.

The average density results are presented in Figure 7.

The density of the obtained composites differed from that of silicone, which is also related to the bulk density of herbal powders—approx. 0.29 g/cm^3^ [48]. The density of pure silicone was 1.08 g/cm^3^, and for the highest fraction, it changed to 1.1 g/cm^3^, so it could be concluded that the density depends on the content of the herbal powders. However, the differences are within the error limits. Statistical significance was found (F (6,28) = 26.86, *p* < 0.001); however, post hoc analysis revealed that the change is only significant in composites with 15 wt.% of thyme and sage. This may be due to the fact that these composites have a higher filler fraction than the rest, and due to the hygroscopic nature of the herbs, the materials are more prone to absorbing moisture, which affects the bulk density. In addition, the content of the woody parts may be an important factor that could potentially influence the standard deviation. The highest standard deviation was observed for composites with higher contents. The presence of moisture is also related to the powders’ structure.

### 3.4. Wettability

**Figure 8:**
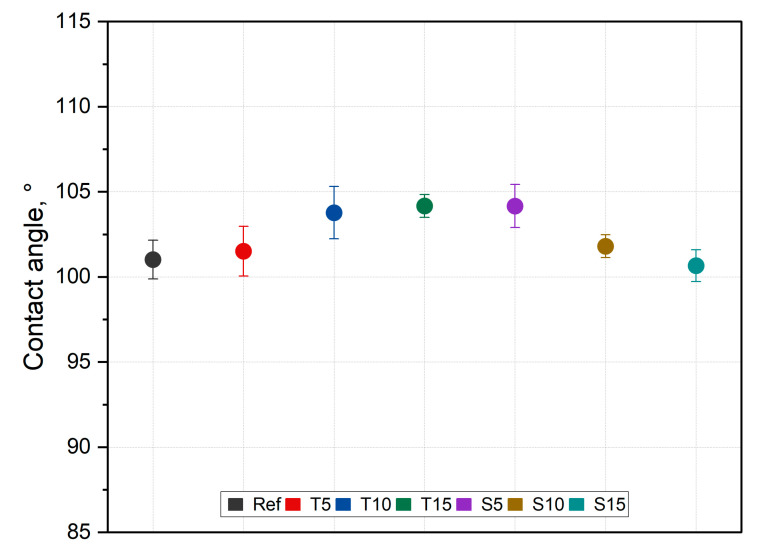
Contact angle results.

The contact angle results are presented in Figure 8.

The incorporation of thyme increases the silicone surface contact angle, and the higher the filler fraction, the bigger the contact angle value. The opposite is observed for sage-filled composites. This is undoubtedly related to the herbal composition (the content of the desired compounds). Sage has a higher acid content than thyme, which affects the nature of the changes in properties (Figure 2). Nevertheless, these changes are within the error limits. Although statistical analysis proved the relevance of the changes (F (6,53) = 3.68, *p* < 0.001); post hoc analysis revealed that the composite incorporated with 15% of thyme alters the contact angle significantly compared to the native material, while the remaining composites’ impact is statistically trivial.

### 3.5. Rebound Resilience

**Figure 9:**
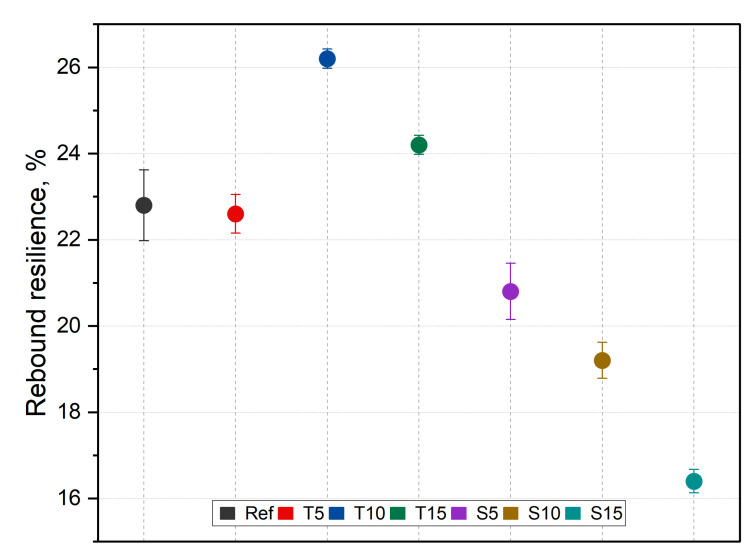
Rebound resilience results.

The rebound resilience results are graphically illustrated in Figure 9.

The resilience of the materials changes depending on the filler type and fraction. The rebound resistance value of T5, T10, and S5 composites is greater than that of silicone. The inverse proportional dependence of rebound resilience on the content is observed for the tested composites, but the values of sage composites are lower than for thyme-filled composites. These differences may be due to the shape of the fillers and the developed surface increasing the moisture absorption, which, in turn, weakens the material. Moreover, sage has a higher crude fat content than thyme, which influences the hardening process of silicone and changes its mechanical properties [37]. The proposed modification of the fillers under the influence of ethanol partially removed the undesirable compounds, e.g., fats. This impacted the fiber and matrix adhesion and the cross-linking process. The most significant decrease is noted for S15 (approx. 19%) compared to the reference samples. One-way ANOVA analysis found the changes to be significant (F (6,28) = 56.26, *p* < 0.001). In relation to the native material, essential changes were noted for T5 and S15.

### 3.6. Antimicrobial Activity Measurements

**Figure 10:**
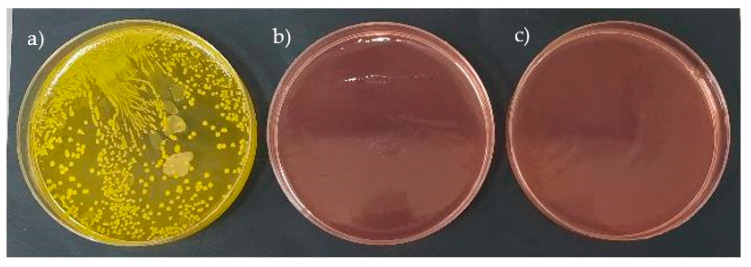
Antibacterial activity results.: reference material—(**a**), T15—(**b**), S15—(**c**).

The results of the antibacterial properties assessment are presented in Figure 10.

It can be noticed that the Gram-positive bacteria have colonized the surface of the reference material. At the same time, the composites exhibited strong antibacterial activity against *S. aureus* after 24 h of incubation. The reduction log for both composites was around 4.4. According to the standard, materials achieving a reduction log bigger than 2 are considered to be bacteriostatic. Similar, yet slightly lower, antibacterial activity was achieved by incorporating zinc oxide nanoparticles [49] and rhamnolipids [50] (two strong antimicrobial agents) into PDMS. Therefore, it is undeniable that the obtained material exhibits bacteriostatic activity against the tested strain. It should be noted that the composites with the highest content of thyme, T15, and sage, S15, were tested because it was assumed that if the matrix material is certified for skin applications, the highest contents will be the most sensitive.

### 3.7. Accelerated Aging

The surfaces of the native and aged samples are presented in Table 4. It should be pointed out that the microscopic observation was performed on the samples after undergoing tensile testing. As can be seen, the surface of the reference material remained the same after aging. In the case of composites, after 2 days of aging, a white deposit is observed on the surface which increases after 60 days of aging. The accelerated aging process conditions led to the precipitation of salts and their adherence to the surface of the samples. This is mainly related to the aging process being carried out in a water-baes salt solution. It should be noted that due to the fact that the studied reference material was translucent, such observation was unfeasible. The solution in which the composites were aged changed its color to light green, indicating that some of the fillers’ compounds dissolved.

### 3.8. ATR-FTIR

**Figure 11:**
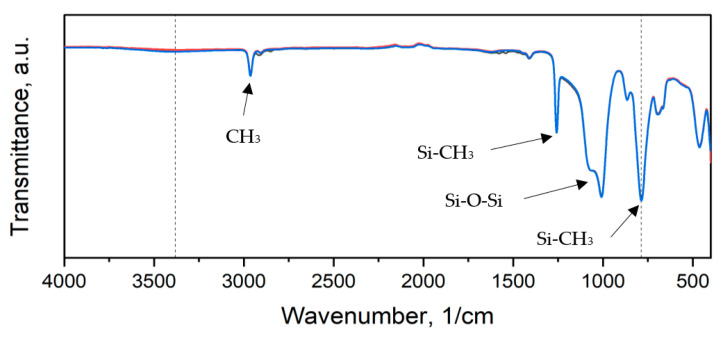
IR spectra of reference material: black—before aging, red—2 days of aging, blue—60 days of aging.

**Figure 12:**
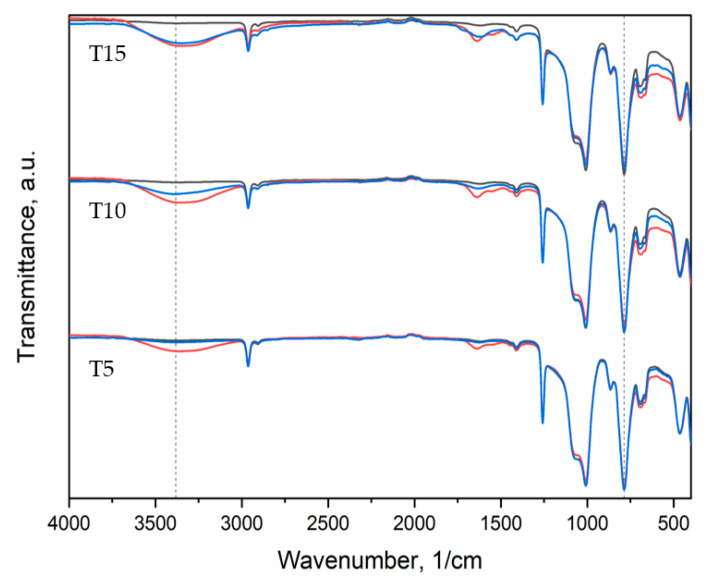
IR spectra of thyme composites: black—before aging, red—2 days of aging, blue—60 days of aging.

**Figure 13:**
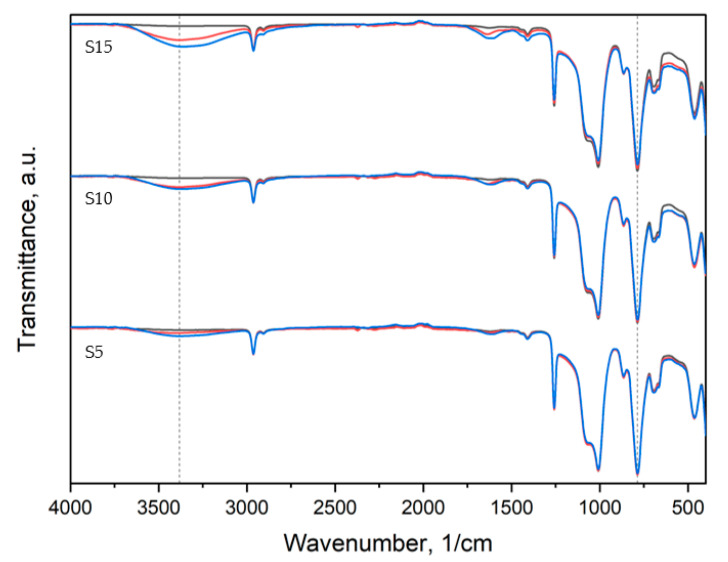
IR spectra of sage composites: black—before aging, red—2 days of aging, blue—60 days of aging.

The IR spectra of the obtained materials are illustrated in Figure 11, Figure 12 and Figure 13.

All samples exhibited peaks corresponding to Si-O-Si, CH_3_ rocking and bending in Si-CH_3_, and C-H stretching in CH_3_ bands characteristic of PDMS [51]. The reference material spectrum remained almost intact after 2 and 60 days of degradation. The peaks corresponding to Si(CH_3_)_2_ stretching and Si-O-Si stretching are of higher intensity for aged samples (Figure S1, supplementary materials).

For thyme and sage composites, the spectrum of the native samples changes with aging days. Peak intensity at 786 cm^−1^ and 1004 cm^−1^ decreases after two days of aging and increases again after 60 days for thyme composites. However, in the case of T5, peaks after 60 days are bigger than in native samples. Similar behavior is observed for the light wave at around 3381 cm^−1^, characteristic of the –OH group (Figure 12). On the other hand, sage composites exhibit decreasing characteristic of PDMS peaks after aging (Figure 13 and Figure S1, supplementary materials). The presence of the hydroxide group in silicones’ chemical backbone decreases the material’s hydrophobicity [52]. As seen in the materials’ FTIR spectra, the fillers’ introduction further decreased the peak corresponding to the hydrophilic hydroxide group (Figure S2, supplementary materials). Therefore, the surface of the composites is more hydrophobic than the reference material. This explains the contact angle results presented in Figure 7. This is crucial from the point of view of biocidal studies, the results of which are presented in Figure 10.

### 3.9. DSC

**Figure 14:**
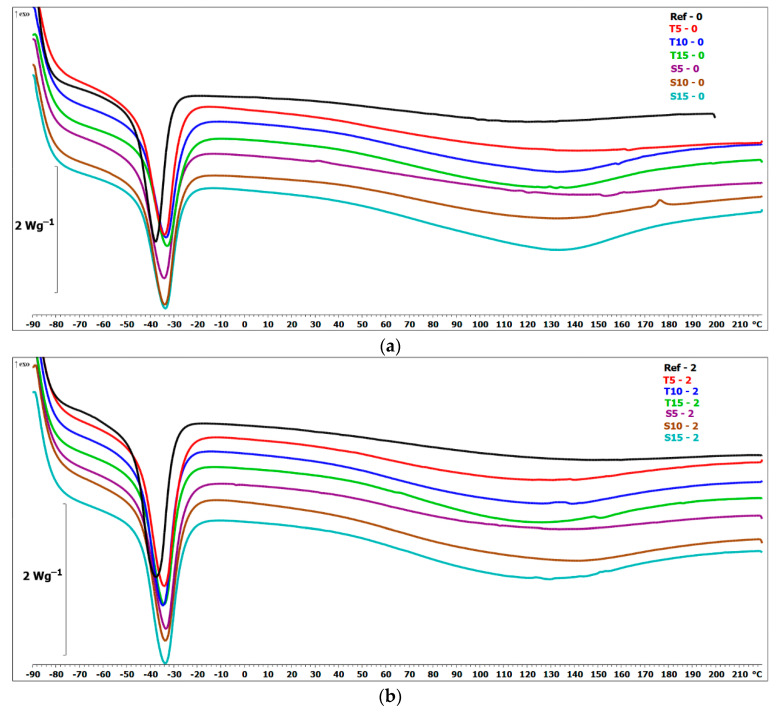

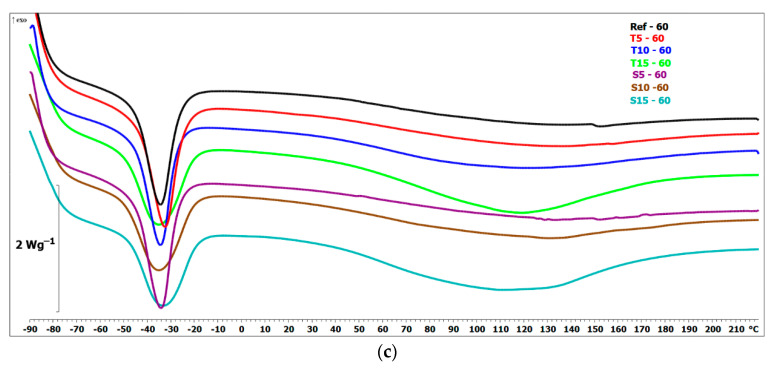
DSC heating thermograms of the tested materials: (**a**) native samples, (**b**) after 2 days of aging, (**c**) after 60 days of aging.

The curing characteristic of the obtained materials is presented in Figure 14, and the results are summarized in Table S3 (supplementary materials).

Based on the data presented, PDMS exhibit an endothermic peak at −40 °C, indicating the melting of the material crystallites, which corresponds to the value reported in the literature [53]. After incorporating thyme and sage, no other endo- or exothermic peak is observed. Moreover, the melting temperatures do not change significantly. The most notable change is observed for T15. There are certain differences between the melting temperature of unaged reference material and biocomposites. However, for the reference samples, after 60 days of aging, the value drops by 3 °C and is comparable to the melting temperatures of the composites. For all composites, the melting point is similar. Similar behavior was observed for the heat of fusion. Adding herbal fillers causes a significant decrease in enthalpy of fusion for composites with 15 wt.% of thyme or sage filler. This effect can be explained by the fact that the presence of the herbal filler particles restricts the nucleation process of polysiloxane. This affects the organization of the crystals leading to the formation of a less crystalline phase within the polysiloxane matrix [54]. Moreover, after 60 days of aging, the enthalpy value slightly decreases compared to the unaged samples. Only in the case of T15 does the value increase after aging. The determined values for individual samples in the first heating run remain almost the same after the second. Accelerated aging had a trivial effect on the tested properties. Nevertheless, adding 15 wt.% of thyme significantly decreased the enthalpy of fusion.

### 3.10. Hardness

**Figure 15:**
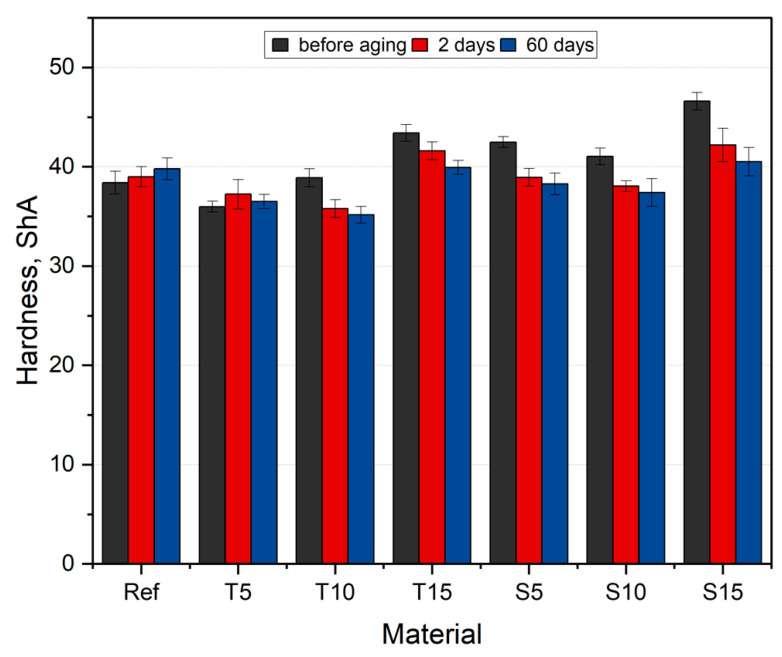
Hardness results.

The hardness average results are illustrated in Figure 15.

All materials exhibit higher hardness than the one stated by the manufacturer (30 ShA). The hardness of the composites has changed compared to the reference group. However, there is no clear relationship between the filler content and type. All samples, except for T5, show a greater hardness value compared to silicone. The biggest increase in value is observed for S15, and the biggest drop in value is observed for T5. An increasing tendency is observed for unaged thyme composites. For T5, the hardness drops by approx. 7%, for T10, it increases by approx. 1%, and for T15, the increase is approx. 11% compared to unmodified material. S5 and S10 composites show a hardness decrease of 3.5% on average, but S15 composites are characterized by a hardness 13% higher than native samples. This is related to the content and distribution of the filler in the matrix, also after the aging period—Figure 15. For all composites, except for T5, hardness decreases with increasing aging days. The biggest decrease is observed for sage composites, where hardness value drops by around 13% after 60 days of aging. The absorbed water-based PBS solution affects the fillers’ homogeneity, confirmed by the FTIR spectra (peak height change around 3381 cm^−1^, characteristic of the -OH group). Hence, smaller series deviations are also observed. As aging progresses under the assumed conditions, the hardness decreases in direct proportion. The longer the aging time, the lower the value. The two-way ANOVA analysis showed a significant influence of both the type and fraction of fillers on the hardness value during aging (*p* < 0.05). Increasing hardness value with aging days can be observed only in the case of reference material.

### 3.11. Tensile Testing

Illustration of the cross-section at the fracture point of thyme and sage composites samples is presented in Table 5. As can be seen, during tensile testing, thyme and sage particles tended to detach and leave voids that acted like notches leading to weakening of the materials. Moreover, the observed changes in topography (Section 3.7) are also observed in the internal structure of the composites.

**Figure 16:**
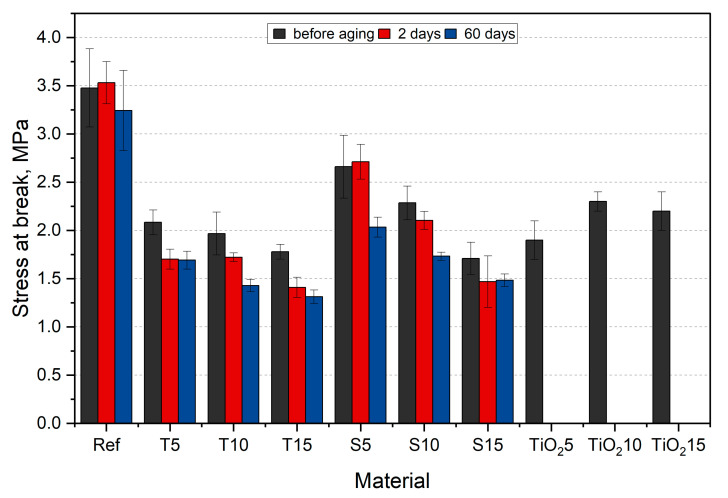
Stress at break results, along with reference data from [55].

**Figure 17:**
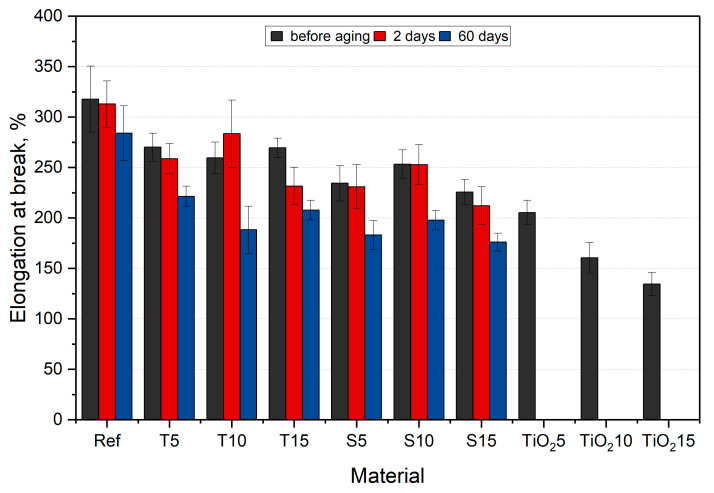
Elongation at break results, along with reference data from [55].

Graphical presentation of the calculated stress at break and elongation at break values are presented in Figure 16 and Figure 17, respectively.

After incorporating organic fillers, the smallest drop in stress at break value was observed for S5 (23%), while the biggest drop in value was observed for S15 (49%). The range of value drop for thyme composites was smaller than that of sage composites (from 40% for T5 to 48% for T15). Such large differences observed for composites with thyme result mainly from the method of preparing herbal powder, in which, apart from soft ground leaves, there are also woody stems (Table 5). Moreover, the properties could be influenced by the agglomeration of the fillers, which act as notches and significantly influence the obtained values. In the case of composites with sage, there is a smaller decrease due to the fact that the leaves and soft stems were selected before the powder preparation. Moreover, as already mentioned, an important factor influencing the tested properties is the herbs’ composition. Similar results were obtained by Mirabedini et al. [55] (presented in Figure 16 and Figure 17 for reference purposes). The researchers incorporated a room-temperature-vulcanizing silicone (RTV) with TiO_2_, an additive that is known to have antimicrobial properties. They observed a decrease in stress at break and elongation at break. The values averaged between 2 and 2.3 MPa correspond to the results obtained in this work. Moreover, Xu et al. [56] reported a study where they obtained an antimicrobial dressing exhibiting similar mechanical properties to ours.

The reference sample’s stress at break increases by approx. 2% after 2 aging days and drops again by 7% after 60 days. Similar behavior is observed for S5 samples. S15 samples after 60 days of aging have the lowest stress at break, dropping by 54% in relation to the aged silicone. The most significant impact of aging on the stress at break value is recorded for T15 samples, where the value drops by 26% after 60 days compared to the unaged samples. The drop in stress at break results after aging corresponds to the decreasing peaks in the materials’ IR spectra.

All materials, except for T10, show a decreasing tendency of elongation at break value with increasing aging days. For native samples, the highest value drop is observed for S15 (29%), while the lowest drop is observed for T5 (15%). For reference, the TiO_2_-filled PDMS exhibited lower elongation at break compared to the studied materials [55]. Among aged composite samples, T10 has the highest elongation at break value, while the lowest is characterized by S15. Sage composites exhibit a similar drop in value after 60 days of aging for all fractions (around 21%). The conducted statistical analysis proved the impact of the filler and aging time on the tensile strength (*p* < 0.05).

As aging progressed, the stress at break decreased, and so did the elongation. However, a smaller scatter of results is observed, which indicates a more homogeneous structure resulting from the influence of the research environment. This is confirmed, as already mentioned, by the FTIR spectra. The absorbed medium affects the fillers by softening them, thus minimizing the influence of more rigid particles.

### 3.12. Multiple-Criteria Analysis

In order to assess the obtained materials, a multiple-criteria analysis (Table 6) was carried out based on the tested properties’ results. The weights were determined based on the influence of the tested characteristics on the operational properties of the materials. The properties, based on their importance, were given a value that ranged from 1 to 7. It should be noted that the antibacterial activity results were not included in the analysis due to the fact that only composites with the highest fraction of fillers were tested.

Based on the multiple-criteria analysis it can be concluded that among the obtained materials, composites with the lowest filler fraction had higher characteristics compared to composites with bigger fraction. S5 had the highest score among the obtained composites.

## 4. Conclusions

The conducted study confirmed the proposed hypothesis that introducing thyme and sage into silicone allows for obtaining antibacterial biocomposites. Within the limitations of this work, the following conclusions can be deduced:

- The inclusion of thyme and sage in various mass fractions influences the chemical, mechanical, and biological properties. This is strongly dependent on the size and morphology of the grain and the chemical composition (acid content)—Figure 2;
- The introduction of the fillers did not significantly change the density and the contact angle. A slight change is observed in the case of density and contact angle measurements. The size and structure of the filler affect the tested properties. Minimal changes in the contact angle of the composite material in relation to the reference samples prove that the modified materials are hydrophobic. However, it should be emphasized that the higher the thyme content, the more the contact angle increases, as opposed to sage;
- Composites with thyme have higher resilience than composites with sage. Moreover, the lower the filler content, the higher the resilience. Adding 5 and 10% of thyme to the matrix positively influences the tested characteristics;
- The developed surface of the fillers influences the absorbency of phosphate-buffered saline (PBS) solution simulating the human extracellular fluid. Significant surface changes are observed after 60 days of aging, as can be seen in Table 4. The higher the filler content, the greater the surface changes after 60 days;
- The hardness, stress at break, and elongation at break decrease with aging time, and the longer the aging time, the smaller the differences between the tested composites, which is caused by the absorption of water by the fillers resulting in a change in their hardness (minimizing the impact of the notch). The results are stable after two days (they do not show surface changes), so they can be used in short-term applications;
- The introduction of fillers does not change silicone’s chemical backbone and melting temperature, regardless of the content. Enthalpy decreases as the filling content increases. During aging, minimal changes in FTIR spectra related to water absorption are observed, and the melting temperature decreases by about 3 °C;
- The applied modification altered the antibacterial properties and exhibited higher efficacy than strong, commonly employed antimicrobial agents;
- Among the obtained composites S5 exhibited the most superior properties based on the multiple-criteria analysis;
- The developed materials can be employed for short-term applications, such as wound dressings or coatings. Further studies will focus on improving the mechanical properties to include high-operational properties demanding applications, i.e., implants.

## Figures and Tables

**Table 1:** Silicone properties [36].

Property	Unit	Value
Density	(g/cm^3^)	1.08
Viscosity	(mPa∙s)	20
Hardness	(ShA)	30
Strain at break	(MPa)	3.45
Elongation at break	(%)	364

**Table 2:** Chemical composition of herbs. Data from [37].

Compound (g/100 g Fresh Weight)	Thyme	Sage
Protein	0.59 ± 0.01	0.62 ± 0.01
Crude fat	1.06 ± 0.12	5.27 ± 0.33
Total carbohydrates	22.30 ± 0.69	18.45 ± 0.39
Ash	2.55 ± 0.14	2.37 ± 0.07

**Table 3:** Materials’ coding and mass concertation.

Filler	Content (%)	Code
Control group		Ref
Thyme	5	T5
	10	T10
	15	T15
Sage	5	S5
	10	S10
	15	S15

**Table 4:** Accelerated aging results.

Material	Aging Days		
	Before Aging	2 Days	60 Days
Ref	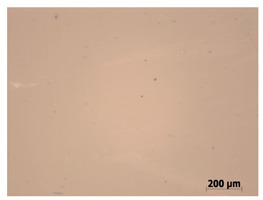	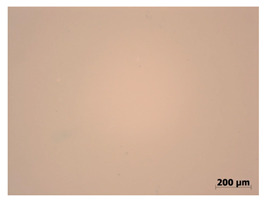	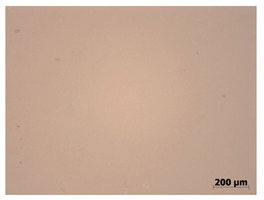
T5	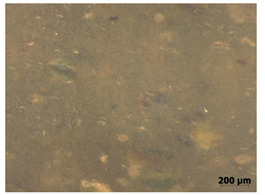	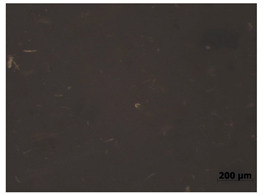	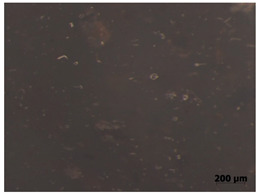
T10	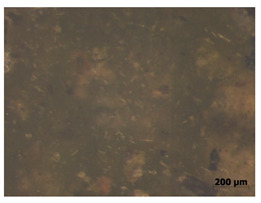	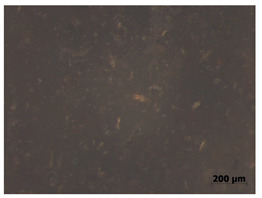	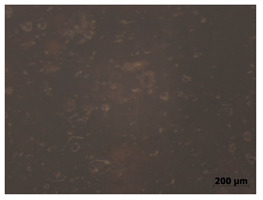
T15	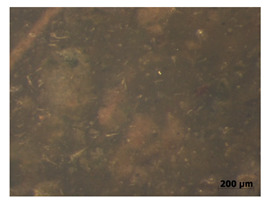	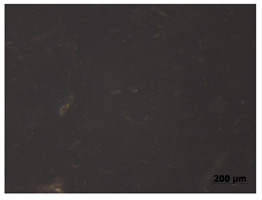	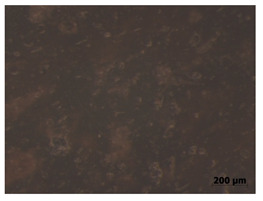
S5	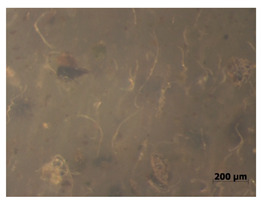	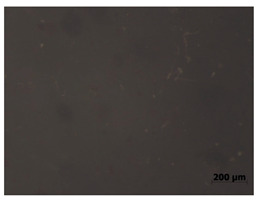	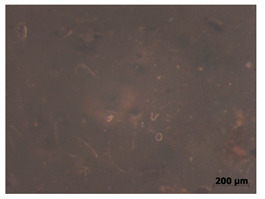
S10	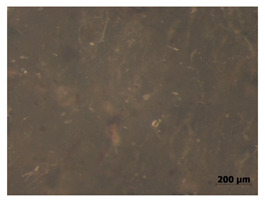	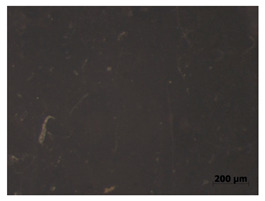	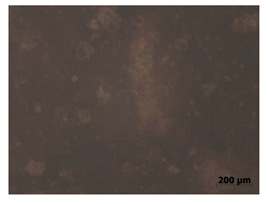
S15	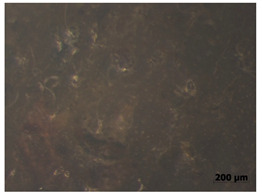	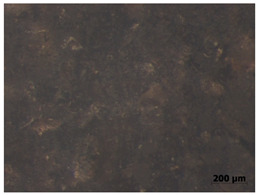	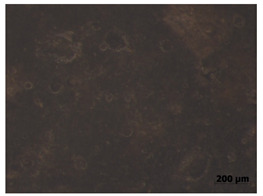

**Table 5:** Cross section of composites’ samples.

Material	Aging Days		
	Before Aging	2 Days	60 Days
T5	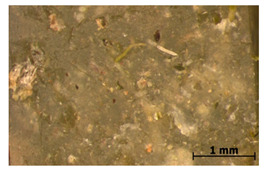	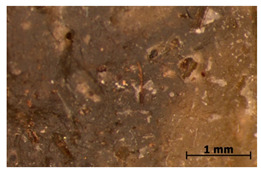	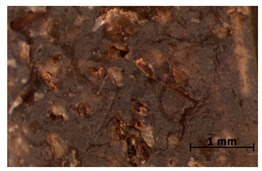
T10	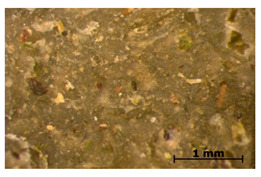	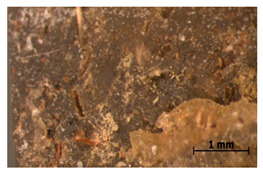	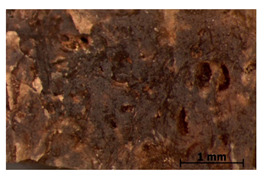
T15	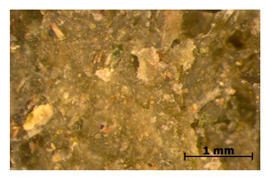	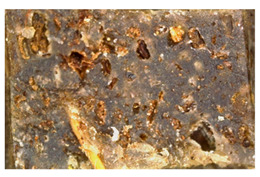	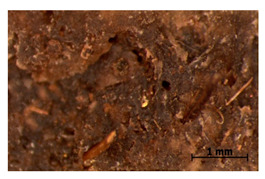
S5	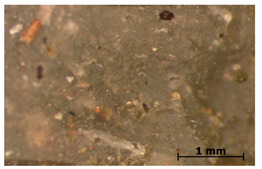	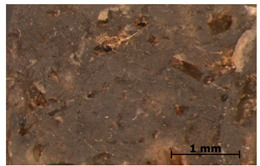	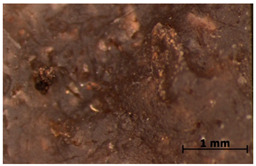
S10	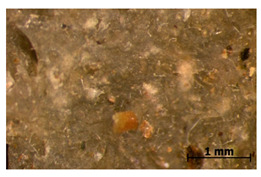	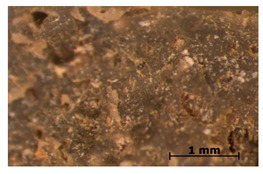	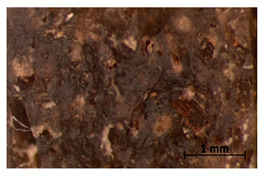
S15	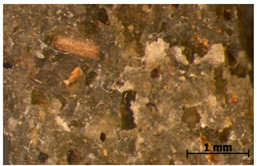	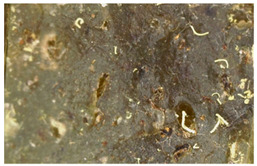	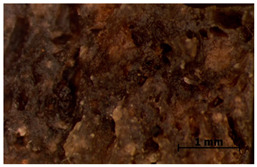

**Table 6:** Multiple-criteria analysis.

Property	Weight	Material													
		Ref		T5		T10		T15		S5		S10		S15	
		C	W	C	W	C	W	C	W	C	W	C	W	C	W
**Density**	1	7	7	7	7	7	7	5	5	6	6	6	6	5	5
**Wettability**	4	2	8	3	12	5	20	7	28	6	24	4	16	1	4
**Rebound resilience**	2	5	10	7	14	6	12	4	8	5	10	3	6	1	2
**Aging impact**	7	7	49	3	21	1	7	2	14	6	42	4	28	5	35
**Hardness**	3	6	18	7	21	5	15	2	6	3	9	4	12	1	3
**Stress at break**	6	7	42	4	24	3	18	2	12	6	36	5	30	1	6
**Elongation at break**	5	7	35	6	30	4	20	5	25	2	10	3	15	1	5
**Σ**			**169**		**129**		**99**		**98**		**137**		**113**		**60**

C—criterion, W—value.

## Data Availability

Not applicable.

## Supplementary Materials

The following supporting information can be downloaded at: https://www.mdpi.com/article/10.3390/polym15010042/s1, Figure S1: IR spectra of the obtained materials in the Si-CH3 stretching band: reference—a, T5—b, T10—c, T15—d, S5—e, S10—f, S15—g; Figure S2: IR spectra of the obtained materials in the –OH stretching band. Table S1. Thermal transitions determined by DSC.
