# Supplementary material for: Influence of Herbal Fillers Addition on Selected Properties of Silicone Subjected to Accelerated Aging
Source: Polymers (Basel). 2022 Dec 22;15(1):42. doi: 10.3390/polym15010042 (PMC9823497; doi:10.3390/polym15010042)
Supplement: Supplementary file 1 [file polymers-15-00042-s001.zip › polymers-2087759-supplementary.pdf]

Supplementary materials

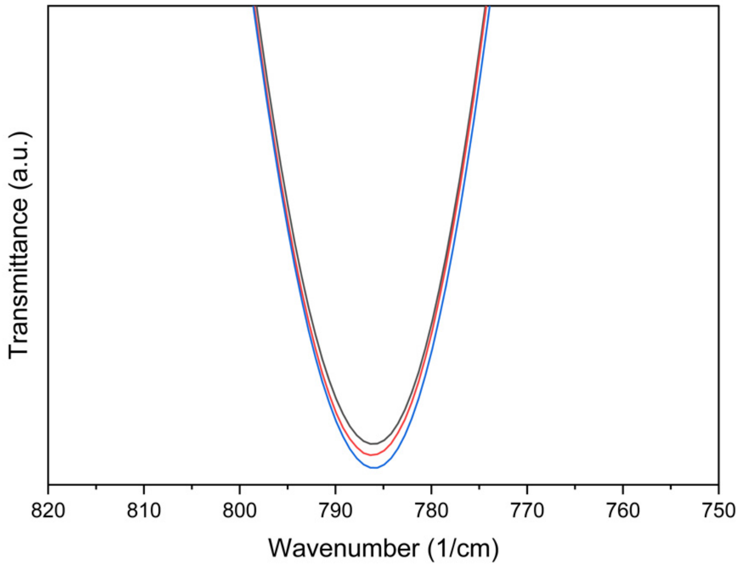

(a)

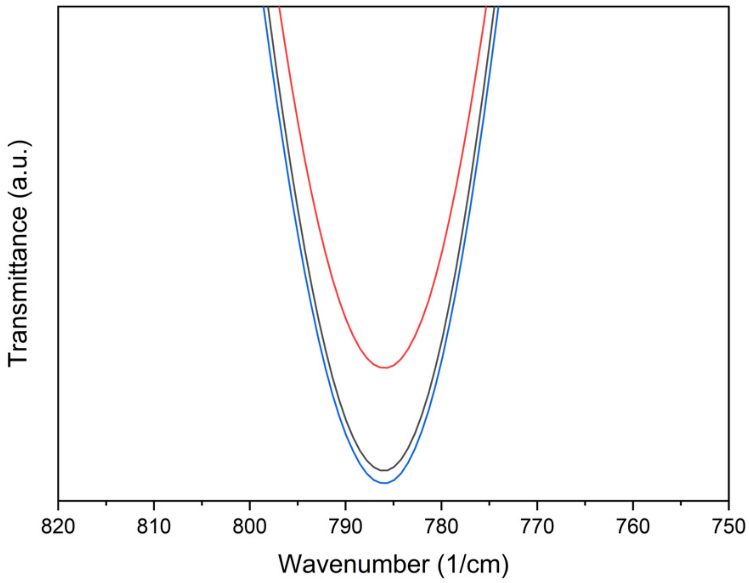

(b)

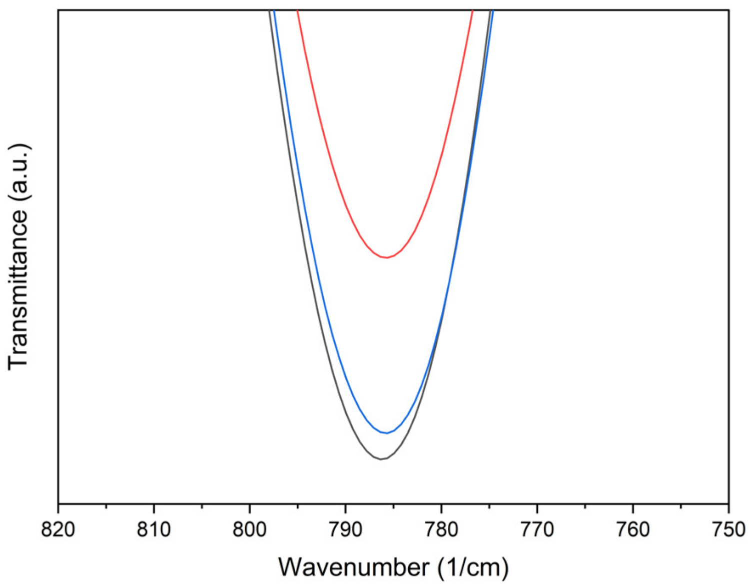
Transmittance (a.u.)
820
810
800
790
780
770
760
750
Wavenumber (1/cm)

(c)

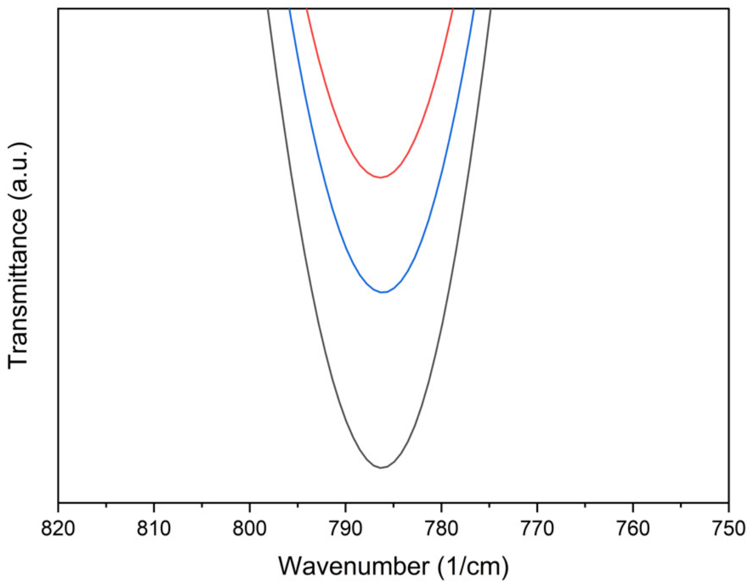
Transmittance (a.u.)
820
810
800
790
780
770
760
750
Wavenumber (1/cm)

(d)

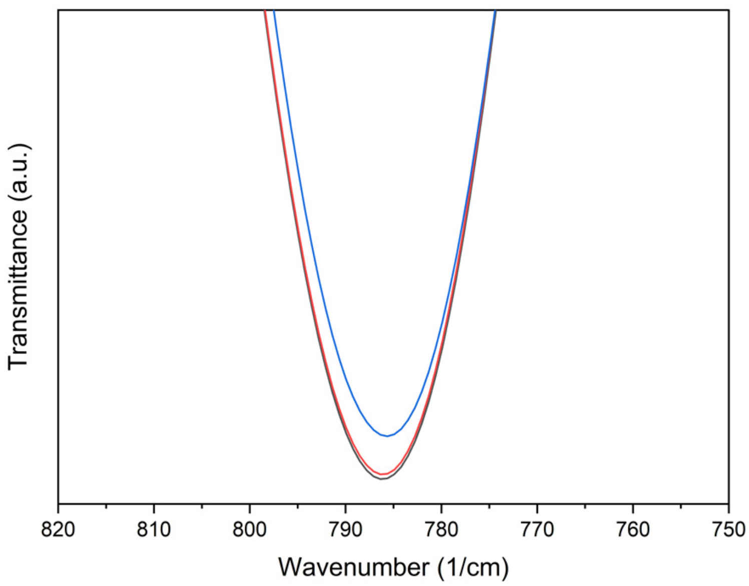
Transmittance (a.u.)
820
810
800
790
780
770
760
750
Wavenumber (1/cm)

(e)

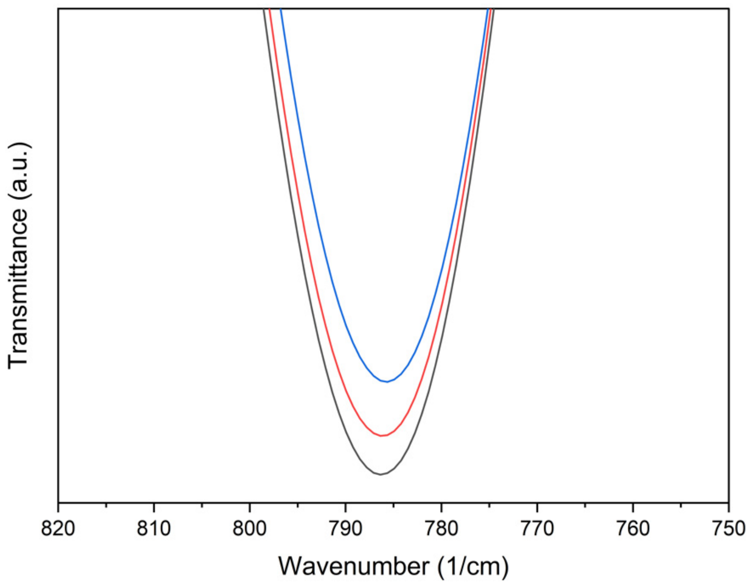
Transmittance (a.u.)
820
810
800
790
780
770
760
750
Wavenumber (1/cm)

(f)

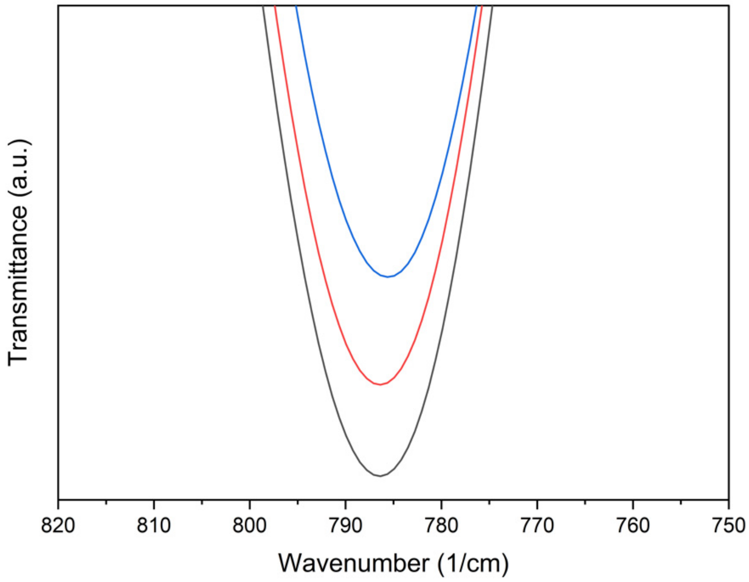

(g)

Figure S1. IR spectra of the obtained materials in the Si-CH_3 stretching band: reference – a, T5 – b, T10 – c, T15 – d, S5 – e, S10 – f, S15 – g.

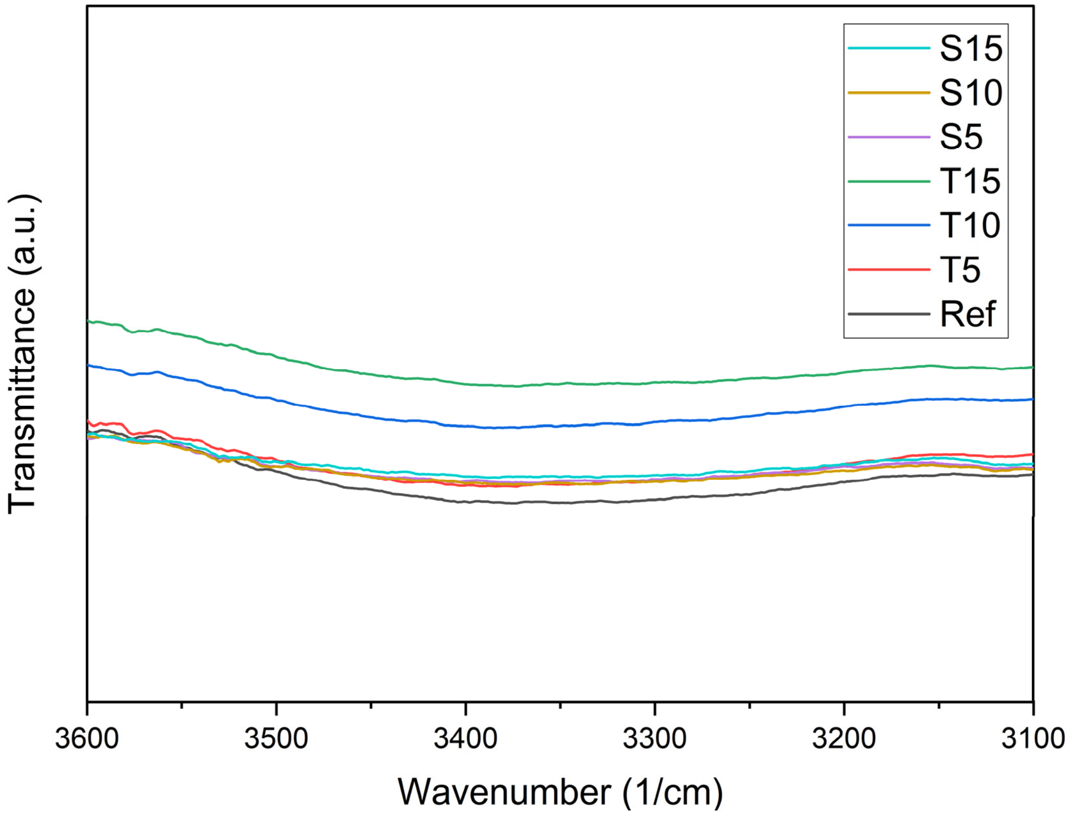

Figure S2. IR spectra of the obtained materials in the –OH stretching band.

Table S1. Thermal transitions determined by DSC.

Sample	I^st heating run				IInd heating run			
	$T_{p,m}$ (°C)	$T_{ei,m}$ (°C)	$T_{ef,m}$ (°C)	ΔH (Jg^{-1})	$T_{p,m}$ (°C)	$T_{ei,m}$ (°C)	$T_{ef,m}$ (°C)	ΔH (Jg^{-1})
Ref	−38.0	−47.4	−31.5	−74.3	−37.6	−47.0	−30.7	−74.3
Ref_2	−38.1	−47.4	−30.3	−76.8	−38.1	−48.1	−30.3	−76.1
Ref_60	−34.7	−45.7	−26.3	−70.9	−35.1	−46.8	−26.6	−71.2
T5	−34.5	−45.4	−27.2	−78.8	−34.5	−45.2	−27.1	−78.4
T5_2	−34.4	−45.4	−26.6	−77.0	−34.4	−45.5	−26.5	−77.0
T5_60	−32.8	−44.3	−24.7	−75.6	−32.8	−43.8	−24.2	−75.4
T10	−33.8	−44.7	−25.9	−71.6	−33.1	−44.1	−25.6	−71.8
T10_2	−35.1	−45.2	−27.9	−73.5	−34.8	−44.6	−27.6	−74.9
T10_60	−34.7	−45.7	−27.3	−69.7	−35.4	−47.4	−27.8	−72.0
T15	−36.4	−45.7	−29.1	−65.5	−35.4	−45.3	−28.2	−65.5
T15_2	−34.0	−44.5	−25.7	−66.5	−34.4	−44.9	−25.9	−66.5
T15_60	−34.4	−45.2	−26.1	−73.5	−34.1	−45.2	−26.0	−72.8
S5	−34.8	−45.5	−26.9	−76.2	−34.5	−45.3	−26.7	−76.2
S5_2	−33.8	−45.0	−26.1	−73.6	−33.4	−45.3	−25.9	−73.9
S5_60	−34.8	−45.5	−26.9	−74.7	−34.1	−45.0	−26.3	−74.8
S10	−34.1	−44.4	−27.1	−74.4	−34.8	−45.0	−27.4	−74.6
S10_2	−34.1	−44.6	−25.9	−72.3	−34.1	−44.7	−26.1	−72.7
S10_60	−34.1	−45.9	−25.9	−69.7	−34.8	−46.5	−27.0	−72.5
S15	−37.1	−46.1	−30.6	−70.1	−36.8	−46.2	−30.4	−70.1
S15_2	−35.7	−46.3	−28.8	−67.4	−36.0	−46.4	−29.3	−67.4
S15_60	−33.1	−43.3	−24.5	−68.1	−33.1	−43.6	−25.0	−68.1
